# Grasping the Alternative: Reaching and Eyegaze Reveal Children’s Processing of Negation

**DOI:** 10.3389/fpsyg.2019.01227

**Published:** 2019-05-24

**Authors:** Alison W. Doyle, Kelsey Friesen, Sarah Reimer, Penny M. Pexman

**Affiliations:** Department of Psychology, University of Calgary, Calgary, AB, Canada

**Keywords:** denial negation, negation processing, eye gaze, visual world, inhibitory control

## Abstract

There is evidence that children begin to understand negation early in the preschool years, but children’s processing of negation is not well understood. We examined children’s processing of denial negation using a variant of the visual world paradigm called the Shopping Task. In this task, participants help a puppet to find the items on a shopping list, selecting from two potential items on each trial in response to the puppet’s affirmative (“the next item is an apple”) or negation (“the next item is not an orange”) sentence. In this binary decision context, participants’ eye gaze and reaching behavior were tracked as they selected the item the puppet wants. Participants were 78 children aged 4–5 years and a comparison group of 30 adults. Results showed that children took longer to process negation than affirmative sentences, and that this difference arose early in processing. Further, children’s eye gaze behavior suggested that on negation trials they regularly looked first to the negated object and were considering the negated meaning early in processing. Adults did not take longer to process negation than affirmative sentences, but their eye gaze behavior also indicated early consideration of negated meanings for negation sentences. We also examined relationships between children’s language and executive function skills and their processing of negation and found no significant relationships. We conclude that both adults and children activate to-be-negated information in the processing of negation. Children, however, are less efficient at processing negation in this context.

## Introduction

Negation is universal to human language, and is commonly used in both adult and child speech, usually in the form of “no,” “not,” and the suffix “-n’t.” Much of the developmental literature has focused on the production of negation (e.g., Klima and Bellugi, 1966; Pea, 1980; Bloom, 1993). This research has documented that many children begin using the word “no” as refusal or non-existence negation around 12 months of age (Dale and Fenson, 1996). Later, around 24–30 months of age, children begin producing denial negation (e.g., “this is not a puppy”), which can be more complex because it typically involves negation of something that was expected or at least plausible. Acquisition of other negative terms continues through the preschool years (e.g., Phillips and Pexman, 2015). Despite its importance to successful communication, there is much we do not yet know about how children come to understand negation. While children’s production of negation is well documented, less research has explored children’s processing of negation. In the present study we investigated children’s processing of denial negation and the developmental skills that might support that processing. We focused specifically on contexts where there are only two (binary) interpretive possibilities for negation: the negated referent and the intended referent.

There is significant debate about the processing mechanism of negation comprehension. Much of the debate concerns whether, in understanding negation, the comprehender needs to activate the to-be-negated information. According to the multi-step processing account (e.g., Wason and Johnson-Laird, 1972; Carpenter and Just, 1975; Dudschig and Kaup, 2018), negation involves mentally representing the negated content or proposition (e.g., a closed cupboard for the sentence “The cupboard is not closed”), followed by its rejection, and finally representing the actual state of affairs (e.g., an open cupboard). By this position, negation will be more difficult to process than affirmative language, because it creates an information processing conflict (Dudschig and Kaup, 2018). In contrast, by the one-step account, it is not necessary that the to-be-negated information first be activated and then rejected when processing negation (e.g., Anderson et al., 2010; Tian et al., 2010; Papeo et al., 2016). The presence of negation markers may activate inhibitory processes that block activation of the negated referent and allow the actual state of affairs to be represented directly (Papeo et al., 2016). Some accounts suggest a third possibility, that the process normally involves multiple steps, except in cases where processing of negation is licensed pragmatically (e.g., Dale and Duran, 2011). In such cases, the negation concept and the target concept could be activated in parallel and eventually bound or fused into a representation for the true state of affairs (Anderson et al., 2010). By this account, one would expect the to-be-negated information to be activated in some contexts but less so in others.

Research on adults’ processing of negation has provided evidence for multiple positions in this theoretical debate. For instance, studies show that adults are slower to respond to negation in picture/sentence verification tasks (Clark and Chase, 1972; Carpenter and Just, 1975). In these tasks, participants judge whether a sentence is a true or false description of a picture. Longer latencies for negated (e.g., “the star isn’t above the plus”) compared to affirmative statements (e.g., “the star is above the plus”) in these tasks are taken as evidence that extra processing is required to understand negation, because the to-be-negated information must first be represented and then recoded. Thus, the findings were taken as evidence for a multi-step account.

The processing of negation was further explicated in a study by Kaup et al. (2006). Kaup et al. (2006) presented affirmative and negated sentences for self-paced reading. Each sentence was followed by a picture at one of two between-subjects delays: 750 or 1500 ms. Participants were asked to name the object in the picture, which either matched or mismatched the state of affairs described in the sentence. At the shorter 750 ms delay, following affirmative sentences (e.g., “the door is open”), participants were faster to name the pictures that matched the actual meaning (i.e., an open door) than the pictures that matched the alternate state (i.e., a closed door). No such difference was observed for negation sentences. However, at the longer 1500 ms delay, following negation sentences (e.g., “the door is not open”), participants were faster to name the pictures that matched the actual meaning (i.e., a closed door) than pictures that matched the negated state (i.e., an open door). The authors interpreted these effects to mean that because participants must first represent the negated state of affairs it takes longer for participants to focus attention on the correct meaning of negation sentences than affirmative sentences; eventually, participants are able to represent only the true state of affairs for negation statements.

In a recent study, Orenes et al. (2014) used a variant of the visual world paradigm (Huettig et al., 2011) with adult participants. Visual world tasks are characterized by the simultaneous presentation of verbal and visual stimuli. During stimulus presentation participants’ eye movements are tracked. Research suggests that when verbal input is received, it is automatically processed and eye gaze shifts toward the visual referent. If negation is presented in the visual world paradigm, it is assumed that participants will tend to look to the referent that is most active at any given moment as they process the negated language. Using this paradigm, Orenes et al. (2014) showed that in binary context (when there are only two possible referents), participants initially fixated on the negated target and then shifted attention to the actual target. This shift in attention took time, and longer latencies were observed for negation than affirmative sentences. Similarly, in a subsequent eyetracking study Orenes et al. (2016) found that negation sentences were processed more slowly than affirmative sentences across several different pragmatic contexts. Thus, they concluded that the to-be-negated information must first be represented and rejected, so negation is always more difficult to process than affirmative language.

In contrast, other adult studies suggest that negation does not necessarily involve representation and rejection of the to-be-negated information and does not always take longer to process than affirmative language. Dale and Duran (2011) used mouse tracking to register responses in a true/false sentence verification task. When sentences were embedded in a context where participants had stronger expectations for negation, the mouse trajectories suggested that equivalent processes were involved for comprehension of negation and affirmative language, and negation did not take longer to process than affirmative language. In contrast, when the sentences were not embedded in strong context mouse trajectories showed evidence for shifts in interpretation during the response process and participants took longer to process negation than affirmative language. Dale and Duran inferred that negation *can* be processed readily when it is licensed pragmatically by context-based expectancies (for ERP data pointing to similar conclusions, see Nieuwland and Kuperberg, 2008). Similarly, Anderson et al. (2010) argued that the actual meaning of negation can be considered from the earliest moments of processing, without necessitating an initial stage where only the to-be-negated information is considered, especially if the negation is used in a situation where there are only binary alternatives.

Thus far, only a handful of studies have examined children’s processing of negation. Kim (1985) showed that 3- to 5-year-old children took longer to process truth-functional negation (e.g., “this is not a car,” presented with a picture of a ball) than true affirmative statements (e.g., “this is a ball”). Nordmeyer and Frank (2014) used eye tracking to examine children’s comprehension of non-existence negation (e.g., “look at the boy with no apples”). Participants were 2- to 4-year-old children and a comparison group of adults. Only the 4-year-old children (and adults) showed good accuracy in their comprehension of negation. In terms of processing, children were less likely to look at the correct target for negated trials than for affirmative trials, but adults did not tend to show this difference. In interpreting their results, Nordmeyer and Frank noted that “… it is possible that when children are presented with two equally likely alternatives, identifying the referent of negation requires ruling out the named object” (p. 36). Thus, children may need to first consider, and rule out, the to-be-negated referent.

Different conclusions were drawn from the eyetracking study described by Reuter et al. (2018). Reuter et al. (2018) investigated processing of “didn’t” by 2- and 3-year-old children. When Reuter et al. provided children with both pragmatic (e.g., story contexts that created expectations for negation) and semantic (blocking of affirmative and negated trials) supports, even 2-year-olds showed above chance accuracy interpreting negation. Further, children showed no differences in processing time for affirmative and negation sentences. Reuter et al. concluded that in pragmatically felicitous contexts it is not necessary for children to first process the to-be-negated meaning in order to understand negation.

Thus, the literature suggests that the processing of negation, for both adults and children, varies across different tasks and discourse contexts. One relevant factor seems to be the presence of context that supports negation. For instance, in adult research, tasks that provide pragmatic support for negation have tended to show equivalent processing times for negation and affirmative statements, leading to the inference that the meanings of negation can be considered directly. Although contextual factors likely explain some of the differences across studies, they do not account for all of the different patterns of results observed. Other relevant factors may include the type of negation, and how processing is measured. For instance, studies that examine only total processing time may miss processing differences between affirmative and negation sentences that could be revealed with other measures, like eye gaze.

Based on the developmental studies conducted thus far, it is not clear which theory is the best description of children’s negation processing, how children’s processing might be different from that of adults, and how children’s processing of negation might be related to their language and cognitive skills. In the present research we approached these questions by investigating how 4- to 5-year-old children and a comparison group of adults process denial negation in a binary context. We used a variant of the visual world paradigm called the Shopping Task that we adapted from Kowatch et al. (2013). By this method, participants evaluate spoken sentences and select real objects based on their evaluations. In our version of the Shopping Task, participants listened to a puppet’s directions about which of two objects the puppet wanted the participant to put in the shopping cart. Across trials, the puppet used both negation (“The next item is not candy”) and affirmative (“The next item is carrots”) sentences to indicate their wants. By age 4–5 years, we expected that children would have high levels of accuracy for comprehension of denial negation (Feiman et al., 2017), and that the focus of our investigation would be their response latencies and eye gaze fixations for correct responses. This method allowed us to measure the extent to which participants considered the to-be-negated meaning (the non-target object), as indicated by their looks to the non-target object for negation vs. affirmative sentences.

We also explored the linguistic and cognitive skills that might be related to children’s processing of negation in order to better understand how negation processing develops. Children with more advanced language skills might be involved in more complex verbal interactions and thus develop more efficient negation processing. Further, inhibitory control involves the ability to reduce or override the influence of a non-target on active processes (for reviews see Diamond, 2013; Petersen et al., 2016) and is an important aspect of a child’s executive functions, along with working memory and cognitive flexibility (Miyake et al., 2000). Such skills have been highlighted as factors that might support negation comprehension (e.g., Nordmeyer and Frank, 2014; Dudschig and Kaup, 2018) and these skills are developing rapidly in the age group we tested (e.g., Davidson et al., 2006; Zelazo and Carlson, 2012). In the present paradigm, inhibitory control and other executive function skills might help children to direct attention away from the negated object and toward the target object and thus may be related to their eye gaze on negation trials. To our knowledge, these hypothesized links between children’s language, cognitive skills, and negation processing have not yet been tested.

## Materials and Methods

### Participants

Participants were 78 children aged 4–5 years (*M* = 61.46 months, SD = 6.43) and a comparison group of 30 adults (*M* = 20.80 years, SD = 2.66). Children were recruited from the University of Calgary Child and Infant Learning and Development (ChILD) database. Children received two small toys as thanks for their participation. Adults were undergraduate students at the University of Calgary, recruited through the Psychology Department subject pool. Adults received bonus credit in a Psychology course in exchange for participation.

### Procedure

#### Shopping Task

We measured participants’ eye gaze and reaction time for negated and affirmative sentences in the Shopping Task. During the experiment participants were seated across a table from the experimenter. There were two toy food objects on the table, one placed on either side of a small toy shopping cart. The experimenter labeled the two food objects when they placed them on the table (“This time we have an apple and an orange”), thus establishing a pair of alternatives. Pete, a puppet worn on the experimenter’s hand, was then introduced and participants were told that their task was to help Pete with his shopping:

Today you are going grocery shopping with Pete the Puppet. Your job is to listen to what Pete says and place the correct item into the basket. Each time Pete talks there will be an item placed on either side of the shopping cart. You’ll need to listen carefully because sometimes Pete will communicate which item he wants by telling you the item he doesn’t want, instead. In other words, sometimes Pete will say, “The next item is a coconut” and sometimes he will say “The next item is *not* a coconut.”

With their hands resting on the table in “ready position,” participants were instructed to listen and to select the object that corresponded to the content of the sentence. There were two practice trials followed by 12 experimental trials: 6 affirmative and 6 negation. Pete’s voice was played on an audio track on a computer. Fifty-four versions of the stimulus materials were created, in order to present each food object as both the target and non-target for both affirmative and negation sentences across all participants and to vary correct target side and trial order.

During study development, a male Canadian English speaker who was naïve to the study purpose recorded the sentence stimuli in a quiet room. The speaker recorded two sentence stems: (1) “the next item is” and (2) “the next item is not,” in order to ensure standardized sentence length up to the point where the food item was named in each sentence. The speaker then recorded the names of the 28 target food items (4 for the practice trials and 24 for the experiment). Using the program Audacity, the name of each food item was added to each of the sentence stems to create the negation and affirmative sentences. The food items were selected from cooking/kitchen toys in pairs that we judged to be of the same food type and of similar desirability for our child participants (e.g., apple and orange, candy and chocolate, see Appendix for full list).

Following the shopping task, three additional cognitive and language measures were presented to the child participants:

#### Red Dog/Blue Dog Stroop-Like Task

We adapted the Red Dog/Blue Dog Task from the “high inhibition” condition of a Stroop-like task described by Beveridge et al. (2002). A line drawing of a cartoon dog was printed in either red or blue fill, then copied onto 26 individual cards such that each card had one red or one blue colored dog on the front. The cards were laminated and stacked in a deck. The researcher held the deck of 26 red dog/blue dog cards, and presented each successive card upon hearing the participant’s previous response, regardless of accuracy. No feedback was provided. The first two cards introduced the dogs: “My name is blue (/red)” was written above the image of the dog. Children were told that the blue-colored dog was called “Red” and the red-colored dog was called “Blue.” Thus the task required that children inhibit the tendency to name the actual color of the dog, and instead use its (opposite) name. The participant was encouraged to say “Hi blue/red” to the first two dogs in order to practice using the name. For the following 24 cards, participants were asked to give the name of the dog upon presentation. The cards were arranged so that participants saw no more than two of the same color of dog in a row.

#### Dimensional Change Card Sort Task

The Dimensional Change Card Sort Task (DCCS) was administered as a measure of cognitive flexibility, following the instructions given in Zelazo (2006). Two small cardboard boxes, identical in size (15 cm × 15 cm × 15 cm) were used as card receptacles, each with a line drawing affixed to the front and back of the box. One box pictured a blue rabbit, the other a red boat. Cards were laminated and cut to 7 cm × 10.75 cm size. The researcher explained the rules of the pre-switch phase, counterbalanced (across participants) to start with either the “color game” or the “shape game.” The researcher gave two example cards, labeling each one by its relevant dimension. For example, “This one is a red one, so it goes in the red box.” Or, “this one is a rabbit, so it goes in the rabbit box.” Participants were asked to sort each of the next six cards into their appropriate boxes, before the post-switch rules were given. To initiate the switch, participants were told “we aren’t going to play the color (/shape) game any more, now we’re going to play the shape (/color) game, where all the rabbits (/blue ones) go in this box, and all the boats (/red ones) go in this box.” Six trials followed in the post-switch phase, and again the researcher labeled each card by its relevant dimension. For example, “here’s a rabbit, where does it go?” All participants completed the pre- and post-switch phase and moved on to the border phase. One example of each type of card (border and no border) was shown to explain the border-phase rules, followed by 12 trials. The border rules were repeated before each of the 12 trials: “if there is a border, we play the color game, and if there isn’t a border, we play the shape game.”

#### Peabody Picture Vocabulary Test

The Peabody Picture Vocabulary Test (PPVT, 4th Edition) was administered as a measure of receptive vocabulary, as outlined in the test manual (Dunn and Dunn, 2007). Participants were familiarized with the procedure through two practice trials. Each test page in the flip book contained four pictures. Participants were shown each test page and when the experimenter named the target word participants were asked to point to the referent. Target words are grouped in sets of 12, and each set gets progressively more difficult. Participants began at set five to establish a basal set in which they made one error or fewer. If the basal set was not achieved at set five, the experimenter went down a set, until the basal set could be determined. The participant’s ceiling set was identified once they made eight or more errors in one set.

### Coding

Each trial was videorecorded via a digital video camera positioned behind the experimenter, with the participant’s face and hands in view. Videos were coded frame-by-frame to assess participant accuracy, reaction time, and eye gaze. For reaction time, children’s responses were divided into three phases corresponding to early, middle, and late processing (Climie and Pexman, 2008; Whalen et al., 2019): lift (onset of object name to initiation of lift), contact (lift to contact with object), and release (contact with object to its release). Thus the coding for reaction time began from the onset of the object name in the puppet’s sentence and ended when the participant released the object into the shopping cart. This method allowed us to consider more than total response time for each trial; the initiation of a physical response at the end of the early phase does indicate that the participant has completed enough processing to begin to respond, but variability could still be observed in later phases of the response and if so we would interpret it as reflective of final verification processes. There were no trials in which a child made contact with the non-target and then altered their reach to grab the target, however, there were several instances in which children initially reached for one item and changed direction. In these cases, the reaction time phases were coded as usual. In addition, children’s eye gaze fixations to the target, non-target, puppet, and extraneous objects were examined. For both affirmative and negation sentence types, eye gaze coding began at the onset of the object name and ended when the participant made physical contact with the object, signifying their choice (the early phases of processing). Eye gaze was coded for whether a participant looked at least once to each of the objects during the total eye gaze coding time and which item participants looked to first (target or non-target) after the object name onset. First look was coded using the method introduced in by Halberda (2006). More specifically, the coder moved forward one frame at a time in the videorecording from the onset of the object name until a participant looked at either the target or non-target (looks to other objects were ignored). Participants were most often looking at the puppets (60% of trials) before they heard the target. On an additional 20% of trials they were looking at the source of the audio (computer speaker) that played the narration. For the remaining 20% of trials, participants were looking at the target or non-target. Thus, for a small proportion of trials participants were already looking at one of the response objects at the onset of the “first look” recording window. We recorded their first look location here even if they were pre-fixated on a response object, since it was impossible in these cases to distinguish intentional from unintentional first looks. First looks to the target were coded as “1” while looks to the non-target were coded as “0”. First look data for 10 child participants were unavailable due to a technical problem with the video files, thus first look was analyzed for 68 child participants (*M* = 60.91 months, SD = 6.75). Trials that were answered incorrectly (1.60%) were excluded from the eye gaze analyses.

A second coder evaluated 25% of the videorecordings (7.5 adult participants and 19.5 child participants). Interrater reliability was assessed using a two-way mixed-model consistency single-measures intraclass correlation coefficient (ICC; McGraw and Wong, 1996; Hallgren, 2012). The resulting ICC was in the excellent range for all variables evaluated (Cicchetti, 1994). For adults: likelihood of looking to target, ICC = 0.92; likelihood of looking to non-target, ICC = 0.91. For children: likelihood of looking to target, ICC = 0.95; likelihood of looking to non-target, ICC = 0.96.

## Results

All Shopping Task data were analyzed at the trial level using mixed effects regressions. Models were computed using the “lme4” package (Bates et al., 2015) in the statistical software R (R Core Team, 2017). All analyses used a maximal linear mixed effect model (Barr et al., 2013) and included random subject and item intercepts as well as by-subject and by-item random slopes for the effect of sentence type and age. We used mixed effects linear regression models to analyze the effect of sentence type (affirmative vs. negation), and for the analysis of children’s data the models included children’s age in months and the interaction of age and sentence type. This way, we could test whether statement type influenced children’s reaching and looking behavior when age was also in the model. In separate regressions we examined whether children’s processing of negation and, separately, affirmative language, was related to the three measures of children’s cognitive/language development: Red/Dog Blue Dog accuracy, DCCS Border Phase accuracy, and PPVT raw score. Mean scores for all child participants on the cognitive and language measures are presented in Table 1. We used the “lmerTest” package (Kuznetsova et al., 2017) to generate *p*-values for models’ fixed effects.

**Table 1:** Mean scores for child participants on the cognitive and language measures.

Measures	Mean	*SD*
Red Dog/Blue Dog Stroop-Like Task	15.88	8.04
Dimensional Change Card Sort Task – Shape	5.55	1.47
Dimensional Change Card Sort Task – Color	5.81	0.97
Dimensional Change Card Sort Task – Border	6.96	2.09
Peabody Picture Vocabulary Test (PPVT)	107.30	17.78

### Adults

#### Reaction Times

**FIGURE 1:**
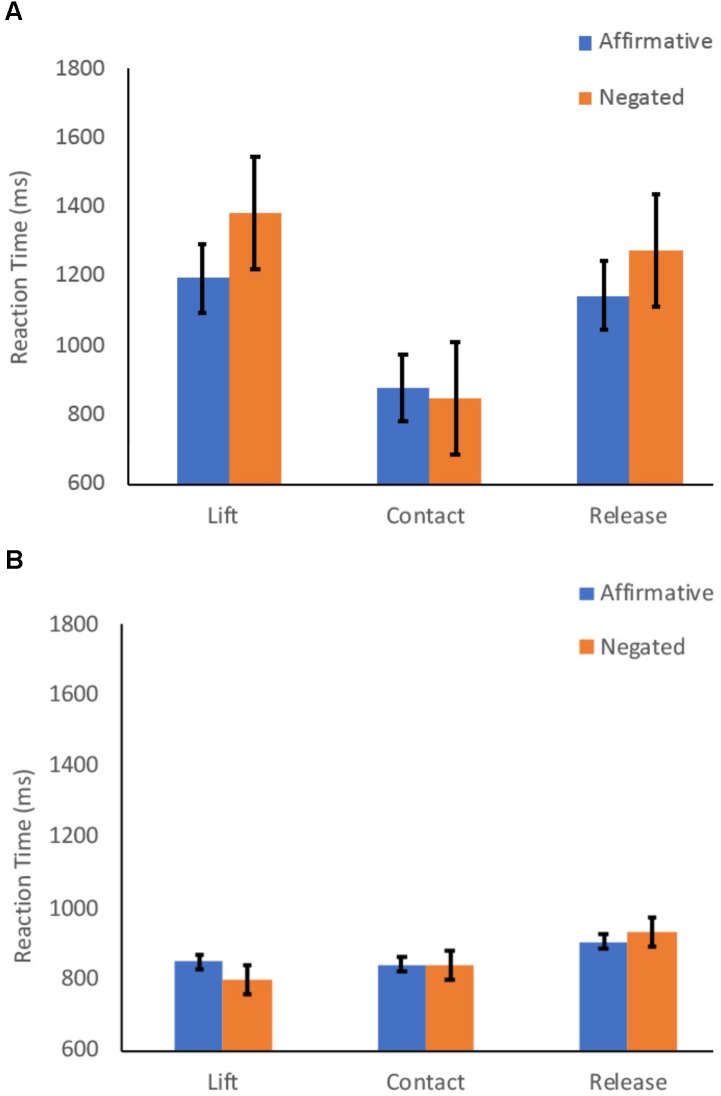
Mean reaction times for **(A)** child and **(B)** adult participants for responses to affirmative and negated sentences in the Shopping Task. Reaching responses were divided into early (lift), middle (contact), and late (release) phases. Bars depict standard errors for mean reaction times in each phase.

We examined adults’ total reaction times and also times for each of the three phases of the response (Figure 1). Results showed that for adults there was no difference in total reaction time for affirmative and negation sentences [β = 14.85 (SE = 40.90), *t* = 0.36, *p* = 0.782]. There were also no differences in reaction times for the early [β = 42.38 (SE = 43.86), *t* = 0.97, *p* = 0.335] and middle [β = 13.96 (SE = 16.46), *t* = 0.85, *p* = 0.402] phases of the response. For the late (release) phase, however, adults were faster for affirmative sentences than negation sentences [β = -41.66 (SE = 15.33), *t* = 2.72, *p* = 0.010].

#### Eye Gaze

Next, we examined adults’ looking behavior during the early phase of processing. Our analyses focused on whether participants looked to the non-target, and which item (target or non-target) they looked to first (see Table 2 for mean likelihood of looking to each of the coded objects for negation and affirmative statements). Results from the logit regression showed that adults were more likely to look to the non-target during negation (*M* = 0.43, SD = 0.50) than affirmative (*M* = 0.28, SD = 0.45) sentences [β = -0.75 (SE = 0.24), *z* = -3.10, *p* = 0.002].

**Table 2:** Mean likelihood of looking to each coded object for adult and child participants (standard deviations in parentheses).

	Adults		Children	
Objects	Negation	Affirmative	Negation	Affirmative
Target	1.00 (0.00)	1.00 (0.00)	1.00 (0.00)	1.00 (0.00)
Non-target	0.43 (0.50)	0.28 (0.45)	0.71 (0.46)	0.58 (0.49)
Extraneous	0.64 (0.48)	0.60 (0.49)	0.94 (0.24)	0.92 (0.27)
Puppet	0.49 (0.50)	0.56 (0.50)	0.58 (0.49)	0.66 (0.47)

For first look, results showed that adults directed their first look to the non-target less than half of the time but did so more often during negation (*M* = 0.38, SD = 0.49) than affirmative (*M* = 0.19, SD = 0.39) sentences [β = 0.53 (SE = 0.13), *z* = 4.14, *p* < 0.001].

### Children

#### Reaction Times

Analyses of children’s total reaction times showed that children were faster to respond to affirmative than negation sentences [β = -129.99 (SE = 55.09), *t* = 2.36, *p* = 0.020]. The effect of children’s age [β = -20.48 (SE = 13.68), *t* = 1.50, *p* = 0.14] was not significant for total reaction times, nor was the interaction of sentence type and age [β = 7.57 (SE = 6.45), *t* = 1.17, *p* = 0.241].

There was also a significant difference in reaction times for the early phase of processing of negation and affirmative sentences [β = -72.61 (SE = 35.55), *t* = 2.04, *p* = 0.043], such that early phase latencies were faster for the affirmative sentences than for the negation sentences. The effect of age was also significant [β = -17.56 (SE = 8.14), *t* = 2.16, *p* = 0.036] such that older age was associated with faster latencies in the early phase of processing. The interaction of sentence type and age [β = 4.07 (SE = 4.19), *t* = 0.97, *p* = 0.334] was not significant.

There was no significant difference in reaction times for affirmative and negation sentences in the middle [β = 11.86 (SE = 18.88), *t* = 0.63, *p* = 0.530] phase of children’s responses. In this analysis, the effect of age [β = -5.92 (SE = 4.32), *t* = 1.37, *p* = 0.173] was also not significant nor was the interaction of age and sentence type [β = 0.11 (SE = 2.88), *t* = 0.04, *p* = 0.969]. There was no significant difference in reaction times for affirmative and negation sentences in the late [β = -57.03 (SE = 44.71), *t* = 1.28, *p* = 0.207] phase of children’s responses. Again, neither the effect of age [β = -10.07 (SE = 7.83), *t* = 1.29, *p* = 0.201] nor the interaction of age and sentence type [β = 5.23 (SE = 5.94), *t* = 0.88, *p* = 0.381] were significant.

#### Eye Gaze

Next, we examined children’s likelihood of looking to the non-target during the early phase of processing. Results of the logit regression showed that children were more likely to look to the non-target for negation (*M* = 0.71, SD = 0.46) than for affirmative (*M* = 0.58, SD = 0.49) sentences [β = -0.29 (SE = 0.08), *z* = -3.64, *p* < 0.001]. The effect of age was not significant [β = -0.012 (SE = 0.013), *z* = -0.91, *p* = 0.363], nor was the interaction of age and sentence type [β = 0.00 (SE = 0.011), *z* = 0.02, *p* = 0.983].

For first look, results showed that children directed more first looks to the non-target for negation (*M* = 0.53, SD = 0.50) than for affirmative (*M* = 0.36, SD = 0.48) sentences [β = 0.324 (SE = 0.08), *z* = -4.11, *p* < 0.001]. In this analysis, neither the effect of age [β = 0.002 (SE = 0.011), *z* = 0.25, *p* = 0.804] nor the interaction of age and sentence type [β = -0.013 (SE = 0.011), *z* = -1.22, *p* = 0.223] were significant.

Finally, we examined whether children’s processing of negation and, separately, affirmative statements was related to their cognitive and language skills. The results are presented in Table 3. Given the number of analyses conducted here, once correction is applied for multiple comparisons, none of the results in Table 3 could be considered significant.

**Table 3:** LME model estimates for the effects of children’s cognitive and language skills on processing of negation and affirmative sentences.

	Negation sentences			Affirmative sentences			Negation sentences			Affirmative sentences		
Fixed effect	Coefficient	Standard error	*p-*value	Coefficient	Standard error	*p-*value	Coefficient	Standard error	*p-*value	Coefficient	Standard error	*p-*value
**Total reaction time**		
Age	-21.43	16.38	0.192	-25.37	24.03	0.295	–	–	–	–	–	–
PPVT	-10.88	8.25	0.192	-9.39	8.49	0.273	-14.33	7.97	0.077	-13.03	7.78	0.098
Red/Blue Dog	1.08	17.74	0.952	-15.83	17.62	0.372	-2.16	17.89	0.904	-18.60	17.43	0.290
DCCS Border	-50.71	68.19	0.460	51.67	68.27	0.452	-52.60	69.43	0.451	42.28	67.74	0.535
**Lift reaction time**												
Age	-3.38	6.59	0.567	-15.48	12.90	0.234	–	–	–	–	–	–
PPVT	-6.65	3.26	0.045	-5.34	4.56	0.246	-7.25	3.11	0.023	-7.56	4.19	0.075
Red/Blue Dog	-9.73	7.00	0.169	-16.61	9.47	0.084	-10.30	6.99	0.145	-18.29	9.39	0.055
DCCS Border	-15.66	26.91	0.563	35.12	36.67	0.342	-15.99	27.12	0.557	29.42	36.48	0.423
**Contact reaction time**												
Age	-5.61	5.42	0.302	-5.55	6.86	0.421	–	–	–	–	–	–
PPVT	0.74	2.33	0.750	0.35	2.43	0.886	-0.16	2.17	0.941	-0.44	2.21	0.842
Red/Blue Dog	0.95	4.91	0.847	2.85	5.02	0.573	0.122	4.88	0.980	2.23	4.95	0.653
DCCS Border	-22.31	18.81	0.239	-22.35	19.52	0.256	-22.77	18.91	0.233	-24.34	19.31	0.212
**Release reaction time**												
Age	-14.22	11.61	0.222	-4.14	12.36	0.739	–	–	–	–	–	–
PPVT	-4.51	5.37	0.403	-4.32	4.37	0.326	-6.80	5.08	0.185	-4.92	3.97	0.220
Red/Blue Dog	9.29	11.43	0.419	-2.48	9.05	0.785	7.14	11.42	0.534	-2.93	8.89	0.743
DCCS Border	-11.07	43.88	0.802	39.41	35.13	0.266	-12.23	44.31	0.783	37.89	34.62	0.277
**Likelihood of looking to non-target**												
Age	-0.0002	0.004	0.961	-0.0004	0.004	0.929	–	–	–	–	–	–
PPVT	-0.002	0.001	0.289	-0.002	0.001	0.246	-0.002	0.001	0.235	-0.002	0.001	0.189
Red/Blue Dog	-0.002	0.003	0.594	-0.006	0.003	0.082	-0.002	0.003	0.581	-0.006	0.003	0.075
DCCS Border	-0.02	0.01	0.071	-0.015	0.012	0.229	-0.023	0.012	0.070	-0.015	0.012	0.218
**First look**												
Age	0.002	0.004	0.603	-0.003	0.005	0.548	–	–	–	–	–	–
PPVT	0.002	0.002	0.307	0.0002	0.002	0.908	0.002	0.002	0.192	-0.0002	0.002	0.917
Red/Blue Dog	-0.002	0.004	0.642	-0.001	0.003	0.699	-0.001	0.003	0.697	-0.002	0.003	0.639
DCCS Border	0.026	0.014	0.068	0.012	0.014	0.264	0.026	0.014	0.066	0.014	0.013	0.295


In the leftmost six columns the analyses include age, whereas in the rightmost six columns the analyses do not include age.

## Discussion

The purpose of the present study was to examine children’s processing of negation. In the Shopping Task, a pair of alternatives was established by the experimenter before the trial started, the speaker used negation on half of the trials, and expectations were created in the instructions that the speaker might communicate what they wanted by stating what they did *not* want. In addition, the task context was binary: on each trial, the speaker referred to one of only two objects.

Results showed that in this context there was evidence that children considered the to-be-negated information when processing negation. This was evident in the eye gaze data. Eye gaze analyses showed that children looked more often at both the target and the non-target for negation sentences than for affirmative sentences. In addition, children were slightly more likely to look first to the non-target than the target on negation trials (53%), and this tendency to look to the non-target first was significantly less common on affirmative trials. These eye gaze data suggest that children often processed the negated meaning before shifting gaze to the correct object. As such, the results could be taken as evidence for the possibility outlined by Nordmeyer and Frank (2014), mentioned here in the Introduction, that children presented with two viable alternatives will need to rule out the named object in order to correctly select the intended referent. In addition, we found that children took longer to process negation than affirmative language. Insights from total processing time can be limited, however, and so further insight was provided by the early phase latencies as these give us clues about what children were considering during early processing. Children’s longer latencies for negation sentences were driven by delays early in processing (the “lift” phase), presumably because it took additional time to activate and then rule out the to-be-negated meaning. Together, these findings suggest that children’s processing was best described by a multi-step account of negation processing.

Adults, too, showed evidence in their eye gaze that they often considered the to-be-negated meaning on negation trials, although perhaps less frequently than did children. Nonetheless, adults still occasionally looked first to the non-target (named object) on negation trials, and did so more often for negation than for affirmative trials. In addition, adults did not take longer to process negation overall, or in the early phases of processing. This suggests that adults were better able to deal with response conflict on negation trials than were children. In contrast, adults showed longer latencies for negation only in the final phase of processing (“release”). This could be taken as evidence for a final integration (fusing) or verification stage that is more time-consuming for negation sentences. As such, on balance, the adults’ data could also be interpreted as consistent with a multi-step account of negation processing. The adult data are also consistent with the notion that there are circumstances where negation can be processed as quickly as affirmative language, such as when it is licensed pragmatically (e.g., Dale and Duran, 2011).

We also explored relationships between children’s cognitive and language skills and their processing of negation, in what we believe is the first examination of this issue. We found no evidence for significant relationships between the measures of children’s inhibitory control or cognitive flexibility and children’s processing of negation. As such, we found no support for the hypothesis that stronger executive function skills might help children to direct attention away from the negated object and toward the target object and thus that these skills would be related to their eye gaze on negation trials. These null findings could be a function of limitations in the present study (e.g., measures chosen), but they do suggest that in the age range tested here other factors may be worth considering in terms of relationships with children’s processing of negation. In addition, it is possible that if younger children were tested then the expected relationships between executive function skills and negation processing might be observed. Younger children would likely find both the Shopping Task and the cognitive assessments to be more challenging and thus their negation processing performance might be more sensitive to individual differences in executive function skills.

There is an extensive literature that has considered the role of alternatives (not just those involved in negation) in language processing. In negation, the use of the word *not* signals to the listener that alternatives should be activated. Given the present task context, there are only two alternatives (the target and the non-target) on each trial and participants likely pre-activate these when they are labeled by the experimenter at the trial’s start. Thus, with the present form of negation there is probably less need for the kind of selection mechanisms that have been described in some of the other work on alternatives, where the listener needs to focus on contextually relevant alternatives, forming an alternative set (e.g., Husband and Ferreira, 2016; Gotzner and Spalek, 2017). The process of considering activated alternatives and suppressing the irrelevant meaning, however, would likely be similar for negation and other types of alternative resolution. One potential difference for negation was identified by Dennison and Schafer (2017). These authors compared adults’ processing of intonationally implicated contrast (e.g., “The mailbox WAS full”) with that of negation (e.g., “The mailbox was not full”). Results showed that the processing time course differed for the two statement types, with negation showing earlier differences in activation of the negated and correct meanings, and contrastive statements showing this difference later in processing. Dennison and Shafer speculated that this could be because the negated meaning, once rejected, does not need to be maintained for understanding ongoing discourse, whereas for contrastive statements the correct and negated meanings both have some relevance for understanding the ongoing discourse.

The results of the present study showed that both children and adults considered the target and non-target meanings early in processing of negation. Adults did not take longer to process negation than affirmative language. Children, however, did take longer to process negation than affirmative language. As such, we infer that while children in the present study were highly accurate at comprehending negation, their processing of negation was not yet as efficient as that of adults. It is possible that adults were better able to make use of the task context, as it licensed negation with speaker knowledge (the speaker description mentioned a tendency to use negation) and a high proportion of negated trials. In future research it will be important to identify the factors that contribute to children’s developing efficiency in processing of negation, and to their emerging ability to draw inferences and derive expectations from the context in which language is used.

## Ethics Statement

This study was carried out in accordance with the recommendations of the Tri-Council Policy Statement: Ethical Conduct for Research Involving Humans, with written informed consent from all adult participants and from caregivers on behalf of child participants. All subjects gave written informed consent in accordance with the Declaration of Helsinki. The protocol was approved by the University of Calgary Conjoint Faculties Research Ethics Board.

## Author Contributions

AD and PP conceived the study. AD, KF, and SR collected the data. AD, KF, and SR analyzed the data. AD, KF, SR, and PP wrote the manuscript.

## Conflict of Interest Statement

The authors declare that the research was conducted in the absence of any commercial or financial relationships that could be construed as a potential conflict of interest.

## APPENDIX

### Practice Trials

(1) Peaches and pineapple
(2) Tuna [in can] and tomato sauce [in can]

### Experimental Trials

(1) Chocolate donut and strawberry donut
(2) [slice of] Cherry pie and [slice of] blueberry pie
(3) Apple juice [in box] and orange juice [in box]
(4) Red pepper and corn on the cob
(5) Candy and chocolate
(6) Vanilla ice cream [on cone] and strawberry ice cream [on cone]
(7) Apple and orange
(8) Peas and carrots
(9) Potato chips and popcorn
(10) Pear and banana
(11) Creamy soup [in can] and vegetable soup [in can]
(12) Watermelon and grapes
